# Social inequalities in health in the Arctic region: from observing to serving

**DOI:** 10.1177/14034948231200515

**Published:** 2023-09-30

**Authors:** Marie Wasmuth Lundblad, Ann Ragnhild Broderstad, Inger Njølstad

**Affiliations:** 1 Department of Community Medicine, UiT The Arctic University of Norway, Norway; 2 Centre of Sami Health Research, UiT The Arctic University of Norway, Norway

The Arctic region includes the northernmost parts of eight countries: Canada, the United States, Russia, Finland, Denmark (Greenland), Iceland, Sweden, and Norway. All these countries are, to different degrees, inhabited by Indigenous populations. The Indigenous populations represent minority groups in the countries in which they reside (except from parts of Canada and Greenland) [1]. They are more exposed to poor living conditions [2, 3] and health outcomes [4] than the majority population, which may be associated with differences in history, culture and development, education and economy, as well as environmental changes in their surroundings.

In the Arctic region we are observing increasing inequalities in health between ethnic groups [5], even in countries where all citizens in principle should have equal access to health services and health literacy [6]. COVID-19 also taught us that ethnic diversity was strongly associated with more severe outcomes (such as hospitalizations and mortality), which could be explained by more crowded living conditions, lack of information, and language barriers among Indigenous population groups.

To combat health inequalities in general, and by ethnic groups in particular, we need a better understanding of the state of health and disease among Indigenous peoples, majority populations, and immigrants. The Arctic region is a particularly important region in this respect, given the heterogeneity of its populations.

In this special issue on Arctic health, we illuminate the health and living conditions of people living in the Arctic region, aspects of their lifestyle (alcohol, diet, and body mass index (BMI)), hypertension, and housing standards.

The COVID-19 pandemic imposed a threat to society, with unknown consequences. This led to emergency responses, where one of the most important measures at the beginning of the outbreak was information about the nature and severity of the disease, and necessary measures. The spread of infection and measures varied enormously between the different countries and areas in the Arctic region. A concern has been raised as to whether Indigenous populations have been properly addressed during the emergency response following the COVID-19 outbreak. Fleury et al. (this issue) showed that 13 out of 16 regions in the northern part of Canada provided information about COVID-19 specific to Indigenous populations. In most cases, this included translation of public health messages into Indigenous languages and forming public health messages to fit Indigenous cultures and daily activities. However, information about infection susceptibility, symptoms, and treatment was mostly missing (Fleury, this issue). In Norway, the COVID-19 pandemic led to tension between local and national authorities, as different measures were implemented in different areas (Fosse, this issue). This was particularly troublesome in rural areas where the lack of medical staff and equipment, in addition to large distances (among others), imposed an additional threat to the local populations (Fosse, this issue). However, measures taken by local chief medical officers and decision makers created more trust in the corresponding local community, both in Norway and Canada (Fosse, Fleury, this issue). The importance and effectiveness of using local decision makers to create and disseminate public health messages should be emphasized in future emergency plans. Therefore, in this special issue we will also examine health outcomes and their determinants in the Arctic regions in light of COVID-19.

The determinants of health from a life course perspective are complex and interrelated. Research has demonstrated that historically, traumatic events and contemporary stressors have an impact on future health and well-being on an individual level, as well as on families and communities. Also, lifestyle factors in the Arctic region are formed by differences between culture and environment. Alcohol consumption, which is a well-known risk factor for health challenges, is shown to be higher in some countries, such as Russia, compared with others, such as Norway [7]. However, Hopstock et al. (this issue) observed that more women and men reported being non-drinkers in Russia compared with Norway, while the proportion of problem drinking was highest in Russian men and in Norwegian women. They also found that problem drinking was more common among the younger generations which might lead to more future challenges caused by alcohol-related problems (Hopstock, this issue). Another important lifestyle factor, particularly for cardiovascular health, is hypertension. Blood pressure and hypertension prevalence is increasing worldwide, the Arctic re-gion being no exception [8]. A study from northern Norway showed that while the prevalence of hypertension was decreasing over time, the treatment and hypertension control measures had increased sixfold (Desai, this issue). As such, the future prevalence of untreated hypertension might be lower, but the burden of treated hypertension will be high, both to individuals and the healthcare system. Other lifestyle factors, including diet, also differ according to history, culture and environment, and although adhering to traditional diets is well acknowledged as healthy (due to less processing of foods), environmental contaminants are of increasing concern in the Arctic. Unguryanu et al. (this issue) observed that the concentration of heavy metals was higher in reindeer (more frequently eaten by Indigenous populations) than in cow, but all levels in both animal species were below the levels regarded as imposing a risk to human health. Overall, food insecurity has been a large challenge for health in Indigenous populations. A report from Canada in 2021 [9] showed that about 5.8 million inhabitants, of whom almost 1.4 million were children below 18 years, were experiencing food insecurity. However, the results from this report did not include information on inhabitants from the territories or Indigenous reserves where food insecurity is known to be high. At the outbreak of the COVID-19 pandemic, large amounts of funds and measures were directed to mitigate the consequences of food insecurity. Horlick et al. (this issue) interviewed inhabitants from Iqaluit and Arviat to create an understanding of how these measures impacted the population. It revealed a large variety of impacts from the COVID-19 pandemic on food security, where the pandemic actually reduced the barriers to food security by, among others, increasing the financial support and thus, more time to harvest.

One of the environmental particularities of the Arctic is the dark winter periods (polar nights) where the sun does not rise at all. This phenomenon is linked to vitamin D deficiency, and in a study from Arkhangelsk, Russia, the median concentrations of vitamin D were lower than recommended in all included age groups (0–60 years), with the lowest values in neonates, followed by school-aged children (7–8 years) and adolescents (13–17 years) (Malyavskaya, this issue). Vitamin D deficiency can have several effects on both physiological and pathophysiological processes and should be carefully monitored in the Arctic region experiencing dark polar nights.

Living conditions and housing in the Arctic differ between countries and municipalities, and between urban and rural areas, and have, correspondingly, varying pertinence to health. In Nunavut, Canada, the Inuit Nunangat area experiences large challenges when it comes to housing of their inhabitants (Sultan, this issue). Overcrowded living conditions and homelessness has led to more respiratory diseases, mental health problems, sexual and physical violence, food insecurity and educational gaps, in addition to upcoming challenges for the younger generations (Sultan, this issue). These are large, important challenges which have become even more relevant in the aftermath of the pandemic. Housing conditions and the area of residence have a large impact on health, and even in areas where the housing conditions are overall of good standards, the impact of area and neighborhood can be observed. In northern Norway, BMI is shown to be linked to area of residence, in that, area–level-of-leisure-time physical activity was inversely associated with BMI (Sari, this issue). This illustrates the importance of improving lifestyles and living conditions at the community level, and not only among individuals.

The findings of this special issue have provided updated and improved knowledge of the causes of suboptimal health in the Arctic region, which should be of importance for public health authorities and policy makers in their search for effective intervention strategies. This includes a better understanding of how culturally relevant interventions are key to improve health and well-being among Indigenous peoples. It is important that the implementation of interventions among these groups involves study participants from the start of planning, up to public dissemination. Participatory research can increase the possibility of participation, increase validity of results, increase generalizability of the findings and attain the trust of the population groups under study.

In terms of the next steps for future research in Arctic research, we would like to stress that the ‘One Health’ approach [10] should be increasingly applied. The ‘One Health’ approach emphasizes that humans, animals, and environment should be viewed as a whole: an interplay of factors closely connected and dependent on each other. This is particularly important when studying populations in the Arctic region, where many have a close connection to animals and nature. Both participatory research and the ‘One Health’ approach should be continuously considered when performing research including populations in the Arctic region.

Finally, we hope that this special issue has provided not only new evidence and guidance for future research in the Arctic area, but that it has served as an inspiration for future research on ethnic inequalities in health. The goal must be to eradicate such inequalities by ensuring equal living standards, access to goods, and healthcare, all of which are necessary prerequisites for the promotion of healthy lifestyles and good health for all.
